# Ressonância Magnética Cardíaca como Ferramenta Diagnóstica Etiológica em Pacientes Recuperados de Morte Súbita Cardíaca ou Arritmias Ventriculares Instáveis

**DOI:** 10.36660/abc.20220411

**Published:** 2023-04-12

**Authors:** Paula C. Marçal, Maria Fernanda Braggion-Santos, Danilo Tadao Wada, Marcel Koenigkam Santos, Henrique Turin Moreira, Gustavo Jardim Volpe, André Schmidt

**Affiliations:** 1 Universidade de São Paulo Faculdade de Medicina de Ribeirão Preto Centro de Cardiologia Ribeirão Preto SP Brasil Universidade de São Paulo Faculdade de Medicina de Ribeirão Preto – Centro de Cardiologia, Ribeirão Preto, SP – Brasil; 2 Hospital das Clínicas Faculdade de Medicina de Ribeirão Preto Departamento de Imagens Médicas, Hematologia e Oncologia Ribeirão Preto SP Brasil Hospital das Clínicas da Faculdade de Medicina de Ribeirão Preto – Departamento de Imagens Médicas, Hematologia e Oncologia, Ribeirão Preto, SP – Brasil

**Keywords:** Morte Súbita Cardíaca, Taquicardia Ventricular Instável, Arritmia Ventricular, Ressonância Magnética Cardíaca

## Abstract

**Fundamento:**

A ressonância magnética cardíaca (RMC) tem relevância diagnóstica crescente em sobreviventes de morte súbita cardíaca (MSC) ou arritmia ventricular instável (AVI) em países desenvolvidos.

**Objetivo:**

Procuramos avaliar retrospectivamente o papel adicional da RMC em um país em desenvolvimento com poucos recursos disponíveis e que pode direcionar um uso mais eficaz desses recursos.

**Métodos:**

Foram incluídos sobreviventes de MSC ou AVI admitidos entre 2009 e 2019 em uma instituição acadêmica terciária após a realização de RMC. Dados demográficos, clínicos e laboratoriais foram coletados dos prontuários. Imagens e laudos de RMC foram analisados e o impacto disso no diagnóstico etiológico final foi afirmado. Realizou-se análise descritiva e definiu-se p<0,05 como significativo.

**Resultados:**

Sessenta e quatro pacientes, 54,9±15,4 anos, sendo 42 (71,9%) do sexo masculino. A maioria dos eventos (81,3%) foi extra-hospitalar e a taquicardia ventricular foi o ritmo mais comum. Medicamentos cardiovasculares foram utilizados anteriormente por 55 pacientes, sendo os betabloqueadores os medicamentos mais utilizados (37,5%). O eletrocardiograma apresentava áreas elétricas inativas em 21,9% e todos apresentavam fibrose na RMC. A média da fração de ejeção do ventrículo esquerdo (FEVE) foi de 44±14%, com 60,9% ≤50% e apenas 29,7% ≤35%. Identificou-se realce tardio com gadolínio em 71,9%, com padrão transmural em 43,8%. A miocardiopatia chagásica foi a etiologia mais comum (28,1%), seguida da miocardiopatia isquêmica (17,2%). Entre 26 sem etiologia previamente identificada, foi possível definir com RMC (15 pacientes - 57%).

**Conclusão:**

De acordo com estudos anteriores em países desenvolvidos, a RMC foi capaz de aumentar o diagnóstico etiológico e identificar o substrato arritmogênico, permitindo melhor atendimento em metade dos pacientes subdiagnosticados.

## Introdução

A morte súbita cardíaca (MSC) é responsável por 53 a 141 eventos por 100.000 pessoas nos Estados Unidos, de acordo com dados consolidados recentes.^1^ A MSC aumenta diretamente com a idade e a doença arterial coronariana (DAC) é a principal causa, responsável por 75%, seguida por outras cardiomiopatias e canalopatias genéticas.^2^ As diretrizes atuais usam a fração de ejeção do ventrículo esquerdo (FEVE) baixa como o principal critério de encaminhamento para a colocação de cardioversor-desfibrilador implantável (CDI) para prevenção primária, e para aqueles que se recuperaram de um evento de MSC ou taquicardia ventricular instável, sendo o CDI indicado como prevenção secundária na maioria das situações se nenhuma causa reversível for evidente.^3 , 4^

Conforme apontado por Myerburg et al., embora uma proporção relativamente alta de eventos de MSC ocorra em pacientes com baixa FEVE, um número significativamente maior de eventos ocorre em pacientes com FEVE preservada.^5^ Estudos epidemiológicos recentes identificaram que a FEVE pode ser um marcador ruim para prevenção primária, uma vez que a maioria dos pacientes que apresentam um evento de MSC não apresenta FEVE baixa. Esses achados reforçam a necessidade de melhores marcadores para minimizar custos e choques desnecessários provenientes do CDI.^6 , 7^

A ressonância magnética cardíaca (RMC) é amplamente reconhecida como uma modalidade de imagem que possibilita informações detalhadas sobre a morfologia, a função ventricular segmentar e global e, principalmente, a caracterização tecidual. Edema e fibrose, por exemplo, são identificados por meio de sequências de imagens específicas. Os padrões de distribuição do realce tardio com gadolínio (RTG) são úteis como ferramentas de diagnóstico, e a literatura vem demonstrando valor prognóstico cada vez maior na identificação de pacientes propensos a MSC em diversas etiologias.^8 - 12^

Poucos estudos demonstraram o valor da RMC na definição etiológica após a recuperação de um evento de MSC.^13^ O uso rotineiro da RMC como parte da avaliação diagnóstica de pacientes com arritmias ventriculares instáveis pode ser desejável em locais com recursos escassos, que precisam ser direcionados de maneira inteligente. Procuramos investigar o valor diagnóstico adicional da RMC de rotina em uma amostra de pacientes com arritmias ventriculares malignas em um país em desenvolvimento.

## Métodos

Realizou-se análise retrospectiva de todos os exames de RMC em um hospital universitário terciário (Hospital das Clínicas da Faculdade de Medicina de Ribeirão Preto da Universidade de São Paulo) no Brasil entre janeiro de 2009 e julho de 2019 para pacientes que apresentaram evento de morte súbita cardíaca abortado ou arritmia ventricular instável. Dados demográficos (sexo, idade), clínicos (cardiopatias prévias, medicações, dados do evento) e laboratoriais foram obtidos a partir de prontuários eletrônicos. A maioria dos exames de RMC ocorreu na primeira internação após estabilização clínica e antes do implante do CDI, quando indicado, com mediana de 26 (IIQ: 10–37) dias.

Os eletrocardiogramas (ECG) feitos durante a primeira internação relacionada ao evento arrítmico e/ou descrição do ritmo durante o evento foram analisados em busca de sinais de fibrose miocárdica definida como onda Q≥0,04 s de duração e ≥25% do tamanho da onda R ou falta de progressão do aumento da onda R nas derivações precordiais e caracterização do ritmo.

Todas as imagens da RMC foram obtidas em um aparelho Achieva 1.5T (Philips Medical Systems, Best, Holanda) com bobina SENSE de 5 elementos (Philips Medical Systems) dedicada a exames cardiológicos. O protocolo incluiu sequências de cine obtidas por precessão livre no estado estacionário (vistas de 2 e 4 câmaras, e uma pilha de 9 a 12 cortes cobrindo ambos os ventrículos no eixo curto), bem como sequências de recuperação de inversão com tau curto ponderadas em T2 utilizando a técnica *black blood* e *turbo spin echo* (TSE) ponderada em T1 em apneia pré-contraste. Posteriormente, os pacientes receberam 0,2 mmol/kg de gadodiamida intravenosa (Omniscan, GE Healthcare, Chicago, Illinois). Após 10 min, adquiriu-se uma sequência inversão-recuperação e gradiente eco rápida para a detecção de RTG nas mesmas posições das cines (vistas de eixo curto, 2 câmaras e 4 câmaras). Os parâmetros dessa sequência foram os seguintes: tempo de repetição, 5,4 ms; tempo de eco, 1,3 ms; ângulo de inclinação, 20°; matriz, 256 × 192; campo de visão, 360 a 400 mm; e espessura do corte, 10 mm (sem lacunas). O tempo de inversão ideal variou de 150 a 280 ms e foi escolhido com base em múltiplos tempos de inversão ( *TI scout* ) imediatamente antes da aquisição RTG.

Todas as imagens foram visualmente analisadas para se obter uma descrição uniforme e a definição do diagnóstico (edema, infiltração gordurosa, presença e padrão de fibrose) por dois revisores cegos para a suspeita clínica e, em caso de discordância, chegava-se a um consenso. Todas as medidas (volumes ventriculares, frações de ejeção ventricular direita [FEVD] e ventricular esquerda [FEVE] e diâmetro diastólico final ventricular esquerdo [DDFVE]) foram extraídas dos laudos dos exames, e os valores normais definidos de acordo com dados de Kawel-Boehm et al.^14^

O estudo foi aprovado pelo comitê de análise institucional (CAEE: 28591920.9.0000.5440) e, devido ao seu desenho retrospectivo, dispensou-se a obtenção de consentimento informado.

### Análise estatística

Por ser um estudo descritivo, as variáveis quantitativas foram descritas como média e desvio padrão ou como mediana e intervalo interquartil quando aplicáveis segundo o teste de Kolmogorov-Smirnov, e as variáveis qualitativas como porcentagens. Utilizou-se o teste Qui-quadrado para avaliar a relação entre a presença de fibrose no ECG e a RMC. Avaliou-se a relação entre as frações de ejeção dos ventrículos direito e esquerdo e a presença de fibrose na RMC. SPSS v.25 (IBM Corporation, EUA) foi o pacote estatístico utilizado, e o nível de significância foi estabelecido em 5%.

## Resultados

### Dados demográficos, clínicos e eletrocardiográficos

Sessenta e oito pacientes preencheram os critérios de inclusão. Quatro pacientes foram excluídos devido à qualidade de imagem inadequada (1 paciente) ou falta de dados clínicos (3 pacientes). Dos 64 pacientes restantes, 42 (71,9%) eram do sexo masculino e a média de idade foi de 54,9±15,4 (16–83) anos.

A maioria dos eventos ocorreu fora do hospital (52 eventos – 81,3%), então 22 (34,3%) foram descritos como paradas cardíacas sem ritmo descrito. Nos 42 pacientes com ritmo identificado, 38 (90%) apresentavam taquicardia ventricular, 2 (5%) apresentavam fibrilação ventricular, um (2%) apresentava *torsades de pointes* e um (2%) apresentava taquicardia de complexo largo.

Cinquenta e três pacientes haviam utilizado medicamentos anteriormente, sendo que betabloqueadores (24 pacientes — 37,5%) e inibidores da enzima conversora de angiotensina ou bloqueadores dos receptores de angiotensina (23 pacientes — 35,9%) foram os mais utilizados. Doze (18,8%) estavam em uso de amiodarona. Durante a internação, o ECG indicou áreas inativas elétricas sugestivas de fibrose em 14 (21,9%) pacientes.

### Ressonância magnética cardíaca

**Figura 1:**
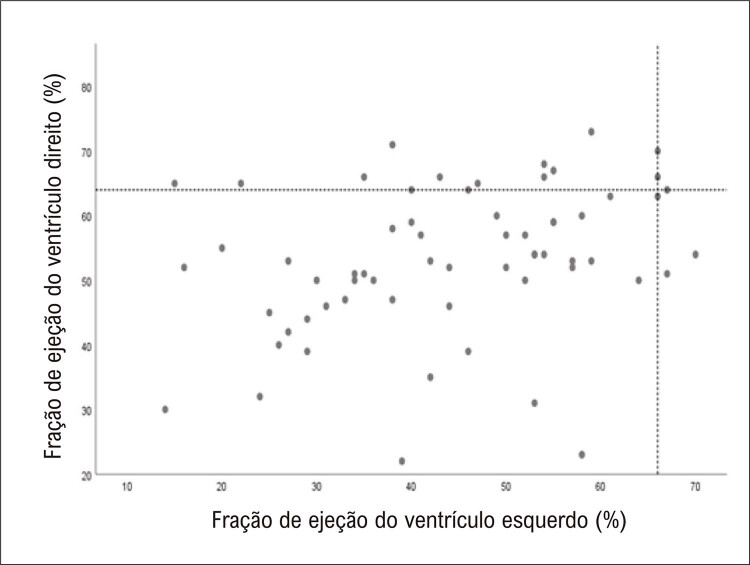
– Gráfico de dispersão entre fração de ejeção do ventrículo direito (FEVD) e fração de ejeção do ventrículo esquerdo (FEVE) com linhas horizontais e verticais definindo os limites normais para cada câmara (66% e 64% para FEVE e FEVD, respectivamente).

A Tabela 1 resume as dimensões ventriculares e as frações de ejeção de ambas as câmaras ventriculares. Apenas 21 (32%) pacientes apresentavam FEVD preservada, e 21 apresentavam ≤50%. O ventrículo esquerdo apresentou fração de ejeção preservada em apenas 9 (14%) pacientes. Considerando aqueles com FEVE reduzida, 39 (60,9%) pacientes apresentavam FEVE ≤50% e, desses, 19 (29,7%) apresentavam FEVE ≤35%. A Figura 1 apresenta a correlação entre FEVD e FEVE em nossa amostra de 64 pacientes avaliados. Como se pode observar, apenas 3 (4,7%) deles apresentaram ambas as frações de ejeção dentro da normalidade.

**Tabela 1:** – Parâmetros volumétricos e funcionais dos ventrículos direito e esquerdo

	Média	Desvio padrão	Mínimo	Máximo
Volume diastólico final indexado do VD (ml/m^2^)	77,3	35,5	21,9	272,3
Volume sistólico final indexado do VE (mL/m^2^)	37,8	28,1	7,3	209,9
Fração de ejeção do VD (%)	53,2	11,4	22,0	73,0
Volume diastólico final indexado do VE (mL/m^2^)	106,7	40,5	35,0	224,6
Volume sistólico final indexado do VE (mL/m^2^)	15,5	128,0	76,6	21,8
Fração de ejeção do VE (%)	44,0	14,0	14,0	70,0

*VD: ventrículo direito; VE: ventrículo esquerdo.*

Identificou-se fibrose em 46 (71,9%) pacientes. Ocorreu padrão transmural em 28 (43,8%), seguido de subepicárdico em 8 (12,5%), médio-miocárdico em 7 (10,9%) e subendocárdico em apenas 3 (4,7%) — Figura Central .

**Figura Central:**
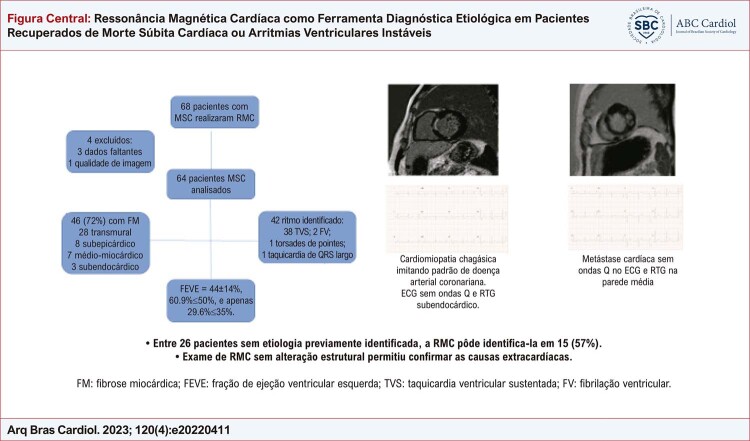
: Ressonância Magnética Cardíaca como Ferramenta Diagnóstica Etiológica em Pacientes Recuperados de Morte Súbita Cardíaca ou Arritmias Ventriculares Instáveis

**Figura 2:**
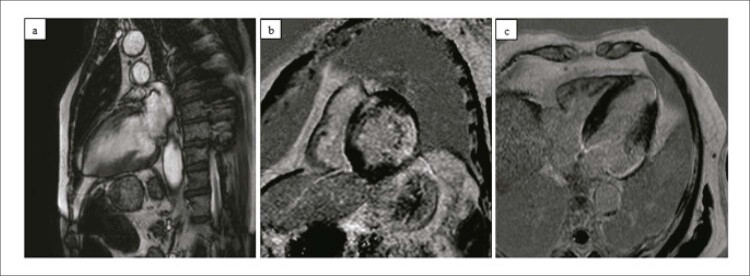
– Paciente com cardiomiopatia chagásica apresentando aneurisma apical na imagem cine (a) e fibrose transmural (b e c) nas sequências de RTG. Também se observa aneurisma apical em c.

A associação entre suspeita de fibrose no ECG e a presente nos exames de RMC foi significativa (Qui-quadrado=0,007) — Tabela 2 . Todos os pacientes com fibrose no ECG (14 pacientes) também apresentaram fibrose na RMC, sendo que em onze era transmural (doença de Chagas ou cardiomiopatia isquêmica) — Figura 2 , dois apresentavam padrão miocárdico médio (cardiotoxicidade e miocárdio não compactado) e em apenas um era epicárdico (displasia arritmogênica). Além disso, a fibrose apresentou associação significativa com uma referência clinicamente utilizada de FEVE baixa (<50%) — Qui-quadrado=0,009. Vale ressaltar que apenas 19 (29,7%) dos 64 pacientes apresentaram FEVE ≤35%. O diagnóstico final após RMC foi variado e está resumido na Tabela 3 .

**Tabela 2:** – Associação de fibrose por ressonância magnética cardíaca identificada em imagens de realce tardio (detecção visual) e presença de fibrose sugerida por ondas q (≥0,04 s de duração e ≥25% do tamanho da onda R ou falta de progressão de aumento da onda R nas derivações precordiais) no eletrocardiograma. Qui-quadrado=0,007

Fibrose	RMC ausente	RMC presente
ECG ausente	18	32
ECG presente	0	14

*RMC: ressonância magnética cardíaca; ECG: eletrocardiograma.*

**Tabela 3:** Diagnóstico etiológico final nos 64 pacientes avaliados

Diagnóstico final	N (%)
Cardiopatia Chagásica	18 (28,1)
Miocardiopatia isquêmica	11 (17,2)
Inconclusivo	7 (10,9)
Cardiomiopatia dilatada	4 (6,3)
Miocardite	3 (4,7)
Diagnóstico misto	3 (4,7)
Cardiomiopatia hipertensiva	2 (3,1)
Cardiomiopatia hipertrófica	2 (3,1)
Displasia arrítmica ventricular	2 (3,1)
Distrofia de Becker	2 (3,1)
Cardiotoxicidade	2 (3,1)
Takotsubo	2 (3,1)
Miocárdio não compactado	1 (1,6)
Metástase cardíaca	1 (1,6)
Outros	4 (6,3)

Com base nas investigações clínicas e laboratoriais iniciais antes do exame de RMC, 26 pacientes não tinham uma etiologia estabelecida. A RMC ajudou a definir a etiologia em 15 (57%) pacientes: 3 (11%) casos de miocardite, 2 (8%) casos de displasia arrítmica ventricular, 2 (8%) casos de distrofia de Becker, 2 (8%) casos de cardiomiopatia hipertensiva, 1 (4%) caso de metástase cardíaca de linfoma de Hodgkin, 1 (4%) caso de miocárdio não compactado, 1 (4%) caso de cardiomiopatia hipertrófica, 1 (4%) caso de cardiomiopatia dilatada familiar e 1 caso de cardiomiopatia de takotsubo (4%). Além disso, um paciente com cardiomiopatia chagásica apresentando realce subendocárdico tardio com gadolínio foi submetido a tomografia computadorizada de coronárias, que identificou obstrução grave da artéria correspondente.

Finalmente, em sete pacientes com diagnóstico inconclusivo na RMC, dois casos foram clinicamente definidos como canalopatias sem cardiopatia estrutural e os outros dois casos tiveram causas não cardíacas (hipocalemia em paciente com doença renal crônica e parada cardíaca durante a indução anestésica). O exame de RMC contribuiu para a confirmação desses diagnósticos ao excluir uma etiologia estrutural, aumentando para 73% sua capacidade de definir o diagnóstico.

## Discussão

O presente estudo indica que a RMC pode desempenhar um papel significativo no estabelecimento da etiologia de um evento de MSC ou arritmia ventricular instável. Sua inclusão no arsenal diagnóstico aprimoraria o tratamento desses pacientes, apresentando uma etiologia ou pela exclusão de doença estrutural, confirmando uma suspeita de causa reversível.

Nossa casuística, em sua maioria homens com eventos ocorridos principalmente em ambiente extra-hospitalar, é semelhante a uma revisão publicada recentemente.^15^ Embora não tenhamos obtido a prevalência de fatores de risco cardiovascular como hipertensão, diabetes, obesidade e tabagismo, 86% faziam uso de algum medicamento relacionado a doenças cardiovasculares, sendo os betabloqueadores os mais utilizados (37,5%).

O ritmo responsável pelo evento foi registrado na maioria dos pacientes (65,5%) e a taquicardia ventricular foi identificada em 90% daqueles com registro de ritmo ou eletrocardiograma realizado durante o evento. Neilan et al. identificaram fibrilação ventricular na maioria dos pacientes avaliados com RMC após um evento de MSC,^16^ concordando com a maioria dos estudos de parada cardíaca súbita extra-hospitalar. Nossa amostra pode fornecer um padrão distinto devido à relevância da cardiomiopatia chagásica no Brasil,^17^ sendo a taquicardia ventricular instável uma causa comum de internação hospitalar devido a essa entidade.^18^

O eletrocardiograma foi capaz de identificar áreas eletroinativas em apenas 14 (21,8%) pacientes, correlacionando com cicatriz na RMC, principalmente quando havia padrão transmural. Estudos anteriores avaliaram a correlação entre os achados da RMC e os achados do ECG relacionados à presença e extensão da fibrose. A principal limitação é a falta de critérios padrão para a definição de cicatriz,^19^ e dados recentes que produziram resultados conflitantes relacionados ao valor das derivações crescentes,^20^ mas o uso de escores de ECG pode ser útil.^21^ Em relação aos pacientes com cardiomiopatia chagásica, parte significativa de nossa amostra, o relato anterior sobre o uso do escore de Selvester é promissor.^22^

Em nossa amostra, assim como em outros estudos,^23 , 24^ a fração de ejeção do ventrículo esquerdo ≤35% esteve presente em apenas 30% dos indivíduos e com 39% acima de 50%, reforçando o conceito de que pode não ser um marcador apropriado de risco de prevenção primária de MSC conforme indicado pelas diretrizes atuais.^25^ Além disso, identificou-se a disfunção ventricular direita em quase um terço de nossa amostra e publicações recentes estabeleceram seu papel central nos acionamentos de CDI e nos eventos de MSC.^26 , 27^ Outra possível explicação para esse achado é a presença de muitos pacientes com cardiomiopatia chagásica em nossa amostra, entidade conhecida por afetar precocemente o ventrículo direito.^28^ Identificou-se realce tardio com gadolínio em 71,9%, da nossa amostra. Esse percentual é quase o dobro do obtido por Rodrigues et al., em coorte semelhante na Inglaterra,^27^ mas semelhante aos obtidos por Iles et al., na Austrália, em coorte de prevenção primária de CDI^29^ e por Neilan et al.^16^ Uma metanálise de 19 estudos de cardiomiopatias isquêmicas e não isquêmicas obteve a confirmação de que o RTG é um importante preditor de arritmias ventriculares em pacientes com insuficiência cardíaca com fração de ejeção reduzida.^30^ O RTG pode ser um marcador independente de prognóstico, conforme demonstrado em outras entidades específicas, como cardiomiopatia hipertrófica,^31^ miocardite^32^ e cardiomiopatia chagásica.^11^ Este último esteve presente de forma significativa em nossa amostra (28,1%), achado esperado devido ao seu padrão de fibrose^33^ e fatores epidemiológicos.

A ressonância magnética cardíaca foi essencial para o diagnóstico em 57% dos 26 pacientes sem diagnóstico definitivo. Estudo anterior em população semelhante verificou que a RMC foi essencial para o diagnóstico em 77% principalmente devido ao padrão de distribuição do RTG, reforçando o valor da caracterização tecidual.^16^ Rodrigues et al. em outra grande amostra de sobreviventes de MSC ou com arritmia ventricular instável, verificaram que a RMC foi essencial para o diagnóstico em 30,4%.^27^ Outra observação importante em nossa amostra é que um exame de RMC sem alterações estruturais permitiu confirmar duas canalopatias e duas causas extracardíacas.

### Limitações do estudo

Nosso estudo apresenta diversas limitações. Primeiro, é um estudo retrospectivo unicêntrico, mas, como outros com desenho semelhante, confirmou o valor adicional da RMC no estabelecimento de um diagnóstico etiológico. Outra limitação é o tamanho da amostra, que pode ser explicado por uma menor sobrevida de indivíduos com MSC devido à falta de equipes de resgate de emergência amplamente disponíveis. Por fim, usamos apenas RTG para caracterização tecidual e novas técnicas, como o mapeamento T1, podem melhorar os recursos da RMC.

## Conclusões

Nosso estudo reforça o conceito de que FEVE baixa não é obrigatória em sobreviventes de MSC e a FEVD pode ser relevante, portanto, sua importância precisa ser mais investigada. A ressonância magnética cardíaca melhorou o diagnóstico etiológico dos sobreviventes de MSC, seja pela identificação de uma causa específica ou pela exclusão de doença estrutural, fornecendo subsídios para intervenções apropriadas para reduzir a morbimortalidade nessa população de alto risco.
