# Supplementary material for: Ressonância Magnética Cardíaca como Ferramenta Diagnóstica Etiológica em Pacientes Recuperados de Morte Súbita Cardíaca ou Arritmias Ventriculares Instáveis
Source: Arq Bras Cardiol. 2023 Apr 12;120(4):e20220411. [Article in Portuguese] doi: 10.36660/abc.20220411 (PMC10263419; doi:10.36660/abc.20220411)
Supplement: Translation

# Translation: Cardiac Magnetic Resonance as an Etiological Diagnosis Tool in Recovered Sudden Cardiac Death or Unstable Ventricular Arrhythmia Patients

**Authors:** Paula C. Marçal, Maria Fernanda Braggion-Santos, Danilo Tadao Wada, Marcel Koenigkam Santos, Henrique Turin Moreira, Gustavo Jardim Volpe, André Schmidt

**Affiliations:** 1 Brasil Universidade de São Paulo Faculdade de Medicina de Ribeirão Preto – Centro de Cardiologia, Ribeirão Preto, SP – Brasil; 2 Brazil Hospital das Clínicas da Faculdade de Medicina de Ribeirão Preto – Departamento de Imagens Médicas, Hematologia e Oncologia, Ribeirão Preto, SP – Brazil

**Keywords:** Sudden Cardiac Death, Unstable Ventricular Tachycardia, Ventricular Arrhythmia, Cardiac Magnetic Resonance

## Abstract

**Background:**

Cardiac magnetic resonance (CMR) has an increasing diagnostic relevance in survivors of sudden cardiac death (SCD) or unstable ventricular arrhythmia (UVA) in developed countries.

**Objective:**

To evaluate retrospectively the additional role of CMR in a developing country where few resources are available, and should be used more effectively.

**Methods:**

The study included SCD or UVA survivors admitted between 2009 and 2019 at a tertiary academic institution referred to CMR. Demographic, clinical, and laboratory data were collected from the medical records. CMR images and reports were reviewed and their impact on the final etiological diagnosis was determined. A descriptive analysis was performed and p<0.05 established as significant.

**Results:**

Sixty-four patients, 54.9±15.4 years old, and 42 (71.9%) males. Most events (81.3%) were out of the hospital and ventricular tachycardia was the most common rhythm. Cardiovascular medications were previously used by 55 patients, and beta-blockers were the most used medications (37.5%). Electrocardiogram had electrical inactive areas in 21.9% and all of them had fibrosis at CMR. Mean left ventricular ejection fraction (LVEF) was 44±14%, with 60.9% ≤50% and only 29.7% ≤35%. Late gadolinium enhancement was identified in 71.9%, with a transmural pattern in 43.8%. Chagas cardiomyopathy was the most common etiology (28.1%), followed by ischemic cardiomyopathy (17.2%). Among 26 without a previously identified etiology, CMR could define it (15 patients – 57%).

**Conclusion:**

In accordance with previous studies in developed countries, CMR was capable of increasing etiological diagnosis and identifying the arrhythmogenic substrate, allowing better care in half of the underdiagnosed patients.

## Introduction

Sudden cardiac death (SCD) is responsible for 53 to 141 events *per* 100.000 persons in the United States according to recent consolidated data.^1^It directly increases with age and coronary artery disease (CAD) is the main cause, responsible for 75%, followed by other cardiomyopathies and genetic channelopathies.^2^Current guidelines use low left ventricular ejection fraction (LVEF) as the main criterion for an implantable cardioverter-defibrillator (ICD) referral for primary prevention, and for those that recovered from a SCD event or unstable ventricular tachycardia, ICD is indicated as secondary prevention in most situations if no reversible cause is evident.^3 , 4^

As pointed out by Meyburg et al., although a relatively high proportion of SCD events occur in patients with low LVEF, significantly more events occur in patients with preserved LVEF.^5^Recent epidemiological studies found that LVEF may be a poor marker for primary prevention, since most patients presenting a SCD event do not have low LVEF. These findings reinforce the need for better markers to minimize costs and unnecessary ICD shocks.^6 , 7^

Cardiac magnetic resonance (CMR) is widely recognized as an imaging modality that allows detailed information about morphology, segmental and global ventricular function, and, particularly, tissue characterization. Edema and fibrosis, for example, are identified by specific imaging sequences. Late gadolinium enhancement (LGE) patterns of distribution are now useful as diagnostic tools, and the literature has been showing prognostic value in identifying patients prone to SCD in various etiologies.^8 - 12^

Few studies have demonstrated the value of CMR in etiological definition after recovery of a SCD event.^13^The routine use of CMR as part of the diagnostic evaluation of patients with unstable ventricular arrhythmias may be desirable in places where resources are scarce and need to be wisely directed. We investigated the additional diagnostic value of routine CMR in a sample of patients presenting malignant ventricular arrhythmias in a developing country.

## Methods

A retrospective analysis of all CMR scans was performed at a tertiary university hospital (Hospital das Clínicas da Faculdade de Medicina de Ribeirão Preto da Universidade de São Paulo) in Brazil between January 2009 and July 2019 for patients presenting an aborted sudden cardiac death event or unstable ventricular arrhythmia. Demographic (gender, age), clinical (previous heart diseases, medications, event data), and laboratory data were obtained from the electronic medical records. Most CMR scans were in the index admission after clinical stabilization and before ICD implantation when indicated with a median of 26 (IQR: 10–37) days.

Electrocardiograms (ECG) during index hospitalization related to the arrhythmic event and/or description of the rhythm during the event were reviewed for signs of myocardial fibrosis defined as Q wave ≥0.04 s in duration and ≥25% of R wave size or lack of progression of R wave increase in precordial leads, and rhythm characterization.

All CMR images were obtained in an Achieva 1.5T scanner (Philips Medical Systems, Best, Netherlands) with a 5-element SENSE coil (Philips Medical Systems) dedicated to cardiologic examinations. The protocol included steady-state free precession cines (2 and 4-chamber views, and a stack of 9 to 12 slices covering both ventricles at the short-axis), as well as black-blood T2-weighted short tau inversion-recovery sequences and pre-contrast Turbo Spin Echo breath-hold T1-weighted imaging. Subsequently, patients received 0.2 mmol/kg of intravenous gadodiamide (Omniscan, GE Healthcare, Chicago, Illinois). After 10 min, a dedicated inversion recovery fast gradient echo sequence was acquired for LGE detection at the same positions as the cines (short-axis, 2-chamber, and 4-chamber views). The parameters of this sequence were as follows: time of repetition, 5.4 ms; time of echo, 1.3 ms; flip angle, 20°; matrix, 256 × 192; field of view, 360 to 400 mm; and slice thickness, 10 mm (no gaps). The optimal inversion time ranged from 150 to 280 ms and was chosen based on a TI-scout scan performed just before the LGE acquisition.

All images were visually reviewed to obtain a uniform description and definition of diagnosis (edema, fat infiltration, presence, and pattern of fibrosis) by two reviewers blinded to the clinical suspicion and a consensus was reached in case of disagreement. All measurements (ventricular volumes, right [RVEF] and left ventricular [LVEF], ejection fractions and end-diastolic left ventricular diameter [LVEDD]) were collected from the reports, and normal values defined according to data from Kawel-Boehm et al.^14^

The study was approved by the institutional review board (CAEE: 28591920.9.0000.5440) and, due to its retrospective design, informed consent was waived.

### Statistical analysis

As a descriptive study, quantitative variables were described as mean and standard deviation or as median and interquartile range when applicable according to the Kolmogorov-Smirnov test, and qualitative variables as percentages. A Chi-square test was used to evaluate the relationship between the presence of fibrosis in ECG and CMR. The relationship between left and right ventricular ejection fractions was evaluated, as well as the presence of fibrosis in CMR. SPSS v.25 (IBM Corporation, USA) was the statistical package used, and the level of significance was established at 5%.

## Results

### Demographic, clinical, and electrocardiographic data

Sixty-eight patients fulfilled the inclusion criteria. Four patients were excluded due to inadequate image quality (1 patient) or missing clinical data (3 patients). Of the remaining 64 patients, 42 (71.9%) were males and the mean age was 54.9±15.4 (16–83) years old.

Most events occurred out of the hospital (52 events — 81.3%) so 22 (34.3%) were described as cardiac arrests with no rhythm described. In the 42 patients with an identified rhythm, 38 (90%) had ventricular tachycardia, 2 (5%) had ventricular fibrillation, one (2%) had *torsades de pointes* , and one (2%) had a wide complex tachycardia.

Medications were previously used by 53 (86%) patients and beta-blockers (24 patients — 37.5%) and angiotensin converting enzyme inhibitors or angiotensin receptor blockers (23 patients — 35.9%) were the most commonly used. Twelve (18.8%) were receiving amiodarone. During hospitalization, ECG indicated electrical inactive areas suggestive of fibrosis in 14 (21.9%) patients.

### Cardiac magnetic resonance

Table 1 summarizes ventricular dimensions and ejection fractions from both ventricular chambers. Only 21 (32%) patients had a preserved RVEF, and 21 had it ≤50%. Only 9 (14%) patients had left ventricle with a preserved ejection fraction. Considering those with reduced LVEF, 39 (60.9%) patients had LVEF ≤50% and, of those, 19 (29.7%) had LVEF ≤35%. Figure 1 presents the correlation of RVEF and LVEF in our sample of 64 evaluated patients. Only 3 (4.7%) of them had both ejection fractions within normal limits.

**Table 1:** – Volumetric and functional parameters of right and left ventricles

	Mean	Standard deviation	Minimum	Maximum
RV indexed end-diastolic volume (mL/m^2^)	77.3	35.5	21.9	272.3
RV indexed end-systolic volume (mL/m^2^)	37.8	28.1	7.3	209.9
RV ejection fraction (%)	53.2	11.4	22.0	73.0
LV indexed end-diastolic volume (mL/m^2^)	106.7	40.5	35.0	224.6
LV indexed end-systolic volume (mL/m^2^)	15.5	128.0	76.6	21.8
LV ejection fraction (%)	44.0	14.0	14.0	70.0

*RV: right ventricle; LV: left ventricle.*

**Figure 1:** – Dispersion plot between right ventricular ejection fraction (RVEF) and left ventricular ejection fraction (LVEF) with horizontal and vertical lines defining the normal limits for each chamber (66% and 64% for LVEF and RVEF, respectively).

Fibrosis was identified in 46 (71.9%) patients. Transmural pattern occurred in 28 (43.8%) followed by subepicardial in 8 (12.5%), mid-myocardial in 7 (10.9%), and subendocardial in only 3 (4.7%) — Central Illustration .

**Central Illustration:** : Cardiac Magnetic Resonance as an Etiological Diagnosis Tool in Recovered Sudden Cardiac Death or Unstable Ventricular Arrhythmia Patients

The association between fibrosis suspected in the ECG and the one present in CMR scans was significant (Chi-square=0.007) — Table 2 . All patients with fibrosis in the ECG (14 patients) had also fibrosis in CMR and in eleven it was transmural (Chagas or ischemic cardiomyopathy) — Figure 2 ; two had a mid-myocardial pattern (cardiotoxicity and non-compacted myocardium) and in only one it was epicardial (arrhythmogenic dysplasia). In addition, fibrosis presented a significant association with a clinically used reference of low LVEF (<50%) — Chi-square =0.009. Noteworthy, only 19 (29.7%) out of the 64 patients had an LVEF ≤35%. The final diagnosis after CMR was diverse and is summarized in Table 3 .

**Table 2:** – Association of cardiac magnetic resonance fibrosis identified in late enhancement images (visual detection) and presence of fibrosis suggested by q waves ( ≥0.04 s in duration and ≥25% of R wave size or lack of progression of R wave increase in precordial leads) in electrocardiogram. Chi-Square=0.007

Fibrosis	CMR absent	CMR present
ECG absent	18	32
ECG present	0	14

*CMR: cardiac magnetic resonance; ECG: electrocardiogram.*

**Figure 2:** – Patient with Chagas cardiomyopathy showing apical aneurysm in cine image (a) and transmural fibrosis (b and c) in LGE sequences. Apical aneurysm is also seen in c.

**Table 3:** – Final etiological diagnosis in the 64 patients evaluated

Final diagnosis	N (%)
Chagas cardiomyopathy	18 (28.1)
Ischemic cardiomyopathy	11 (17.2)
Inconclusive	7 (10.9)
Dilated cardiomyopathy	4 (6.3)
Myocarditis	3 (4.7)
Mixed diagnosis	3 (4.7)
Hypertensive cardiomyopathy	2 (3.1)
Hypertrophic cardiomyopathy	2 (3.1)
Ventricular Arrhythmic dysplasia	2 (3.1)
Becker dystrophy	2 (3.1)
Cardiotoxicity	2 (3.1)
Takotsubo	2 (3.1)
Non-compacted myocardium	1 (1.6)
Cardiac metastasis	1 (1.6)
Other	4 (6.3)

Based on the initial clinical and laboratory investigations before the CMR scan, 26 patients did not have an established etiology. CMR helped define the etiology in 15 (57%) patients: 3 (11%) myocarditis, 2 (8%) ventricular arrhythmic dysplasia, two (8%) cases of Becker dystrophy, two (8%) hypertensive cardiomyopathies, one (4%) cardiac metastasis from a Hodgkin’s lymphoma, one (4%) non-compacted myocardium, one (4%) hypertrophic cardiomyopathy, one (4%) familial dilated cardiomyopathy, and Takotsubo (4%). Besides, one Chagas cardiomyopathy patient presenting subendocardial late gadolinium enhancement was submitted to coronary computed tomography scan and a severe obstruction of the corresponding artery was identified.

Finally, in seven patients with inconclusive diagnosis at CMR, two cases were clinically defined as channelopathies without structural heart disease, and the other two cases had non-cardiac causes (hypokalemia in a chronic kidney disease patient and a cardiac arrest during anesthesia induction). CMR contributed to confirming those diagnoses by ruling out a structural etiology, increasing its ability to define diagnosis to 73%.

## Discussion

The present study indicates that CMR may play a significant role in establishing the etiology of a SCD event or unstable ventricular arrhythmia. Its inclusion in the diagnostic armamentarium would refine the treatment of those patients by providing an etiology or, by ruling out structural disease, confirming a suspected reversible cause.

Our sample, mostly with men and with events occurring mainly out of the hospital, is similar to a recently published review.^15^Although we did not obtain the prevalence of cardiovascular risk factors such as hypertension, diabetes, obesity and smoking, 86% were using some cardiovascular disease-related medication, and beta-blockers were the most used ones (37.5%).

The rhythm responsible for the event was registered in most patients (65.5%), and ventricular tachycardia was identified in 90% of those with a rhythm strip or ECG during the event. Neilan et al., identified ventricular fibrillation in most patients evaluated with CMR after a SCD event,^16^in accordance with most studies of out-of-hospital sudden cardiac arrest. Our sample may provide a distinct pattern due to the relevance of Chagas cardiomyopathy in Brazil,^17^and unstable ventricular tachycardia is a common cause of hospital admission due to this entity.^18^

Electrocardiogram was able to identify electroinactive areas in only 14 (21.8%) patients correlating with a scar in CMR, especially when a transmural pattern was present. Previous studies evaluated the correlation between CMR findings and ECG findings related to the presence and extension of fibrosis. The main limitation is the lack of standard definition criteria of scar,^19^and recent data provided conflicting results related to the value of increasing leads^20^but the use of ECG scores may be useful.^21^Concerning Chagas cardiomyopathy patients, a significant portion of our sample, previous report on the use of the Selvester score is promising.^22^

In our sample, as in other studies,^23 , 24^left ventricular ejection fraction ≤35% was present in only 30% of the subjects and with 39% above 50%, reinforcing the concept that it may not be an appropriate marker of SCD primary prevention risk as indicated by current guidelines.^25^In addition, right ventricular dysfunction was identified in nearly one-third of our sample and recent publications established its central role in ICD firings and SCD events.^26 , 27^Another possible explanation for this finding is the presence of many Chagas cardiomyopathy patients in our sample, an entity known to affect early the right ventricle.^28^Late gadolinium enhancement was identified in 71.9% of our sample. This percentage is nearly double that obtained by Rodrigues et al., in a similar cohort in England,^27^but similar to those obtained by Iles et al., in Australia, in an ICD primary prevention cohort,^29^and by Neilan et al.^16^A metanalysis of 19 studies of ischemic and non-ischemic cardiomyopathies obtained a confirmation that LGE is an important predictor of ventricular arrhythmias in patients with heart failure with reduced ejection fraction.^30^LGE may be an independent marker of prognosis as demonstrated in other specific entities like hypertrophic cardiomyopathy,^31^myocarditis,^32^and Chagas cardiomyopathy.^11^The latter was significantly present in our sample (28.1%), an expected finding due to its fibrosis pattern^33^and epidemiological factors.

Cardiac magnetic resonance was essential for diagnosis in 57% of those 26 patients without a definite diagnosis. A previous study in a similar population found that CMR was essential for diagnosis in 77% mainly due to the LGE distribution pattern, reinforcing the value of tissue characterization.^16^Rodrigues et al. (2017), in another large sample of survivors of SCD or with unstable ventricular arrhythmia, found CMR to be essential for diagnosis in 30.4%.^27^Another important observation in our sample is that a CMR scan with no structural abnormality allowed confirming two channelopathies and two extracardiac causes.

### Study limitations

Our study has several limitations. First, it is a retrospective unicentric study but, like others with a similar design, it confirmed the additional value of CMR in establishing an etiological diagnosis. Another limitation is the sample size that may be explained to a lower survival rate of SCD individuals due to the lack of widely available emergency rescue teams. Finally, we used only LGE for tissue characterization and new techniques such as T1 mapping may improve CMR capabilities.

## Conclusions

Our study reinforces the concept that low LVEF is not mandatory in SCD survivors and RVEF may be relevant, so its importance needs further investigation. Cardiac magnetic resonance improved the etiological diagnosis of SCD survivors, either by identifying a specific cause or by ruling out structural disease, providing support to appropriate interventions to reduce morbidity and mortality in this high-risk population.
